# The carbon footprint of arthroscopic procedures

**DOI:** 10.1308/rcsann.2023.0036

**Published:** 2023-06-29

**Authors:** S Shah, H Morris, N Nicolaou, S MacInnes, P Haslam, S Shahane, F Ali, J Garcia

**Affiliations:** ^1^ Sheffield Teaching Hospitals NHS Foundation Trust, UK; ^2^ Doncaster and Bassetlaw Teaching Hospitals NHS Foundation Trust, UK; ^3^ East Midlands North Training Rotation, UK; ^4^ Sheffield Children’s NHS Foundation Trust, UK; ^5^ Chesterfield Royal Hospital NHS Foundation Trust, UK

**Keywords:** Sustainability, Environment‌, Orthopaedics, Arthroscopy

## Abstract

**Introduction:**

The healthcare sector contributes the equivalent of 4.4% of global net emissions to the climate carbon footprint; between 20% and 70% of healthcare waste originates from a hospital's operating theatre and up to 90% of waste is sent for costly and unneeded hazardous waste processing. This study aimed to quantify the amount and type of waste produced during an arthroscopic anterior cruciate ligament reconstruction (ACLR) and an arthroscopic rotator cuff repair (RCR), calculate the carbon footprint and assess the cost of the waste disposal.

**Methods:**

The amount of waste generated from ACLR and RCR procedures was calculated across a range of hospital sites. The waste was separated primarily into clean and contaminated, paper or plastic. Both carbon footprint and cost of disposal across the hospital sites was subsequently calculated.

**Results:**

RCR generated 3.3–15.5kg of plastic waste and 0.9–2.3kg of paper waste. ACLR generated 2.4–9.6kg of plastic waste and 1.1–1.6kg of paper waste. The cost to process waste varies widely between hospital sites, waste disposal contractors and method of waste disposal. The annual burden of the included hospital sites for the arthroscopic procedures undertaken was 6.2 tonnes of carbon dioxide.

**Conclusions:**

The data collected demonstrated a significant variability in waste production and cost for waste disposal between hospital sites. At a national level, consideration should be given to the procurement of appropriate products such that waste can be efficiently recycled or disposed of by environmentally sustainable methods.

## Introduction

The health sector’s mission is to promote and protect health. However, it makes a major contribution to the greatest health threat of the 21^st^ century, the climate crisis. Climate change has been labelled as ‘the biggest global health threat of the 21^st^ century’.^1^ The climate footprint of the healthcare sector is equivalent to 4.4% of global net emissions, and if it were a country, would be the fifth-largest emitter on the planet.^2^

In 2018, the Intergovernmental Panel on Climate Change announced that to limit global warming to 1.5°C, greenhouse gas emissions must decrease 45% by 2030 compared with 2010 and reach net zero by 2050.^3^ In the United Kingdom, Public Health England and the National Health Service (NHS) have estimated the health and social care climate footprint within England in 2017 to be around 6.3% of the country’s climate footprint.^2^

The United Nations Sustainable Development Goals strive to protect the planet and end poverty by 2030. These goals emphasise the need to strengthen health systems by building service-delivery capacity and ensuring sustainability; they have already been addressed by the surgical community, but attention has been paid to service delivery as opposed to waste management.^4^ Adequate hospital waste management contributes to success in several of the goals; in particular, health and wellbeing, clean water and sanitation, decent work and economic growth, responsible consumption and production, and climate action.^5^

The Health Technical Memorandum for the safe management of healthcare waste, published in 2013, provides an update to that previous published in 2006.^6^ It has been suggested that between 20% and 70% of healthcare waste originates from a hospital’s operating theatre. Up to 90% of operating theatre waste is improperly sorted and sent for costly and unneeded hazardous waste processing.^7^ Between individual hospitals and trusts, there is still significant variation in the way in which healthcare waste is disposed of which, in turn, affects the cost of waste disposal and the environmental burden.

There is a lack of published data on the proportion of waste disposed via the different waste streams, yet the choice of waste stream has up to a 50-fold impact on a procedure’s carbon footprint.^8,9^ The purpose of this study was to quantify the amount and type of waste produced during two common orthopaedic arthroscopic operations: arthroscopic rotator cuff repair (RCR) and arthroscopic anterior cruciate ligament reconstruction (ACLR). Initially undertaken in a single unit, subsequent data were captured from surrounding hospitals that highlighted the variability in practice.

## Methods

Prospective data collection was undertaken. All procedures were undertaken by senior surgeons.

Five each of ACLR and RCR from each centre were included. Clinical waste created by anaesthetic colleagues was excluded.

Hospital site A is a district general hospital that uses reusable gowns and disposable drapes. Hospital site B is a district general hospital utilising disposable drapes and gowns. Hospital sites C and D are specialist tertiary level centres that also utilise disposable drapes and gowns. It is worth noting that one of the hospital sites, site A, used reusable tray packs, drapes and gowns, and did not use disposable options.

Intraoperative waste from ACLR and RCR procedures was measured. The waste was separated into four bags: clean paper, contaminated paper, clean plastic and contaminated plastic. Contaminated waste is any that has been used directly in patient care; it does not include product wrappers but would include each item in a multipack surgical set, even if the item had not been used. At the end of each procedure, each bag of waste was weighed using a theatre scale DIGI^®^ DS-502 (Marsden Weighing Machine Group Ltd, Rotherham, UK). The mean measurement of waste was taken for each procedure from each centre.

Subsequently, the waste management policy of each hospital was requested alongside the tariff for waste management and carbon dioxide (CO_2_) emissions for different types of waste from each waste management contractor. Each waste management contractor is obliged to provide data on carbon emissions, cost and the way in which they process the waste. The cost and carbon emissions for processing each type of waste were calculated for each centre.

Finally, a calculation was undertaken based on the annual surgical figures (before the COVID-19 pandemic) from the hospital sites to calculate their annual burden of CO_2_ emissions for ACLR and RCR surgeries using the data on carbon emissions provided by the waste contractor.

## Results

### Overall waste data

**Figure 1:**
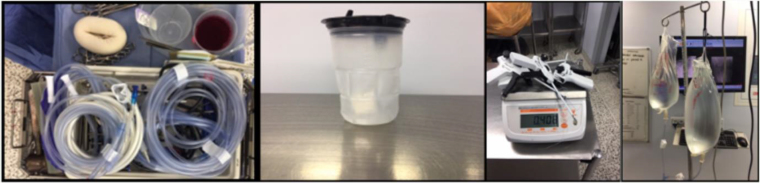
Suction tubing (0.31kg), suction bag (0.1kg), plastic disposable medical devices and plastic saline bags (0.84kg)

Table 1 demonstrates the range of total waste across the hospital sites and the mean total weight of clean and contaminated paper and plastic waste across the hospital sites. Figure 1 shows commonly disposed plastic items and their weights.

**Table 1:** Mean weight of clean and contaminated waste across hospital sites per procedure

Procedure	Paper waste (kg)			Plastic waste (kg)		
	Total	Clean	Contaminated	Total	Clean	Contaminated
ACLR	1.1–1.6	1.1	0.28	2.4–9.6	3.3	2.9
RCR	0.9–2.3	0.9	0.45	3.3–15.5	2.2	4.7

ACLR = arthroscopic anterior cruciate ligament reconstruction; RCR = rotator cuff repair

### Individual hospital sites

Table 2 and Table 3 demonstrate the mean plastic and paper waste generated across individual hospital sites for an ACLR and RCR, respectively.

**Table 2:** Mean plastic and paper waste across individual hospital sites per arthroscopic anterior cruciate ligament reconstruction procedure

Hospital	Textiles	Clean waste (kg)	Contaminated waste (kg)	Total plastic waste (kg)
A	Reusable gowns, disposable drapes	1.0	2.1	2.4
B	Disposable	1.4	3.5	3.6
C	Disposable	5.4	5.6	9.6

**Table 3:** Mean plastic and paper waste across individual hospital sites per rotator cuff repair procedure

Hospital	Textiles	Clean waste (kg)	Contaminated waste (kg)	Total plastic waste (kg)
A	Reusable gowns, disposable drapes	1.2	3.4	3.5
B	Disposable	4.2	10.3	13
D	Disposable	2.3	13.2	14.7

### Carbon emissions and processing costs

Each hospital has a contract for waste management that is negotiated on an individual basis.

• Hospital A pays £124 per tonne for the management of clean recyclable waste and £215 per tonne for the management of contaminated recyclable waste. The contract involves the use of specific waste bags that allow identification and sorting such that contaminated waste is incinerated and the energy released during this process is harvested and reused.
• Hospital B pays £264 per tonne for the management of clean recyclable waste and £714 per tonne for the management of contaminated recyclable waste. The contaminated waste is disposed of into landfill.
• Hospitals C and D pay £103 per tonne for the management of clean recyclable waste and £301 per tonne for the management of contaminated recyclable waste. Contaminated waste is disposed of into landfill.

Despite the hospitals separating out clean plastic and paper waste, none of the hospital waste contractors provide a recycling service and so this waste is incinerated with energy recapture. Of the contaminated waste, waste was either disposed of into landfill or by incineration. The carbon emissions of processing a tonne of waste via these methods were taken from information provided by each waste contractor. Subsequently, the carbon emissions were calculated using these figures.

Table 4 and Table 5 demonstrate the mean total waste per ACLR and RCR procedure, respectively, for each individual site, the cost of processing this and the carbon emissions produced.

**Table 4:** Mean waste, processing costs and carbon emissions for 1,000 arthroscopic anterior cruciate ligament reconstruction procedures across individual hospital sites

Hospital	Textiles	Total waste (kg)	Carbon emissions (tonnes)	Processing costs (£)
A	Reusable gowns, disposable drapes	3,100	0.34	949
B	Disposable	4,900	0.87	2,842
C	Disposable	11,000	2.6	2,668

**Table 5:** Mean waste, processing costs and carbon emissions for 1000 rotator cuff repair procedures across individual hospital sites

Hospital	Textiles	Total waste (kg)	Carbon emissions (tonnes)	Processing costs (£)
A	Reusable gowns, disposable drapes	4,600	0.53	880
B	Disposable	14,500	2.6	8,463
D	Disposable	15,500	6.4	4,210

### National figures

Pre-COVID-19, the hospital sites had an annual burden for ACLR of 7.53 tonnes of clinical waste generated and 1.7 tonnes of carbon dioxide. For RCR surgeries, the annual burden from the hospital sites was 15.4 tonnes of clinical waste generated and 4.5 tonnes of carbon dioxide. The higher proportion of plastic waste in RCR surgeries generated the proportionally larger carbon footprint.

To put this into context, flying from London Heathrow to New York John F Kennedy airport generates 986kg of carbon dioxide. The combined annual contribution for arthroscopic ACLR and RCR surgeries at these three sites is the equivalent of 6.3 return flights.^10^

## Discussion

In the Health Technical Memorandum, clinical waste is defined as ‘ … any waste which unless rendered safe may prove hazardous to any person coming into contact with it … and any other waste arising from medical practice.’^6^ Healthcare waste is divided into non-hazardous and hazardous waste and is strictly controlled with regulations; for European Union Member States, this includes healthcare waste classification from Annex III of Directive 2008/98/EC and a List of Waste established by Commission Decision 2014/955/EU.^11,12^

The main components of healthcare waste are plastic (39.3%–50%), textile (14%–31%), paper (11.2%–25%), glass (0.3%–22.7%), woodware (3.2%–20%), rubber (3.4%–6.6%), metal (0.3%–5%) and other waste (1.4%–18.6%).^13^

The World Health Organization (WHO) estimates that 80%–85% of all healthcare waste is non-hazardous and 15%–20% is hazardous.^14^ It has been estimated that over half of the global population is at risk from environmental, occupational or public health threats resulting from improperly treated healthcare waste.^15^ Improper healthcare waste management may occur for a variety of reasons including a lack of awareness about health hazards from healthcare waste, inadequate training in proper waste management, lack of infrastructure or energy, lack of appropriate regulations or a failure to enforce existing regulations.^16^ To enable appropriate waste care management, the WHO has made recommendations to separate healthcare waste into different coloured trash bags with labels. These then undergo a range of disposal methods depending upon their contents. Recyclable waste should be recycled, whereas contaminated waste undergoes processing by incineration, with or without energy-harvesting methods, or by landfill. Single-use metals, disposed of in a sharps bin, are processed by incineration.^6^

### Reducing waste and application of a circular economy to healthcare

There are several ways in which to reduce clinical waste; for example, applying a circular healthcare economy model: reducing, reusing, recycling.

• Reducing what is consumed can be achieved by identifying whether the item needs to be opened for the set. Do we need to use multipack options of surgical tools when single-packed items may suffice? Reducing the amount of plastic used is critical because the recycling and processing of clean plastic waste generates a greater carbon footprint than the processing of clean paper waste, even though the financial cost to the trust was the same in the contracts we analysed.^17^
• Reusing items by selecting items that can be laundered reduces the production and subsequent consumption of items; for example, the use of reusable kidney bowls, light handles and drapes. Our data suggest that 7kg of waste is from drapes and gowns for an ACLR or RCR. Given that a recent review could not support disposables as more effective in orthopaedic or spinal surgery in reducing surgical site infection, as professionals we should be questioning whether we are making appropriate decisions with the surgical textiles we are using.^18^
• Recycling allows for a reduction in waste going to incineration and landfill.
• If waste has to be disposed of, then utilising ways in which energy can be harvested and reused is preferable to landfill.

The clean waste generated by an arthroscopy includes the wrappers of items such as drapes, giving sets and saline bags; effectively things that are wasted before any patient contact. The remainder of the waste is deemed clinical waste because it is involved in direct patient care. In the NHS, clinical waste is hazardous waste unless it is from a municipal source not in any way associated with healthcare (e.g. cosmetic body art or piercing) or it is segregated non-cytotoxic and non-cytostatic medicine.^6^

Hazardous clinical waste can be broadly classified into one of three categories: that containing an infectious substance, that which is a chemical hazard or that containing a pharmaceutically active agent.^6^ The authors would challenge whether more consideration should be given to the guidance for disposal. For example, for the waste to be deemed infectious, when applied to arthroscopy, the main concern is that of contamination from synovial fluids and the potential risk of infection from these. Interestingly, the same guidance counts sanitary waste including incontinence pads as non-infectious and non-clinical waste unless the patient has a documented infection such as *Clostridium difficile*^6^ yet these potentially pose a far greater risk of infection transmission given the nature of faeces.

The NHS guidance suggests that medicinal waste includes discarded items contaminated with medicinals, such as connecting tubing or drug vials.^6^ The saline used to infiltrate the joint during arthroscopy is not a medicinal product and would pose little harm to an individual during disposal if not classified as medicinal waste. The implication of this is that the saline bags and connecting tubing could then be recycled as clean, not contaminated waste, thus increasing the amount of waste for recycling and reducing the quantity for disposal by incineration or landfill.

If further consideration was given to the national guidelines, it is likely that significantly more items (e.g. giving sets, connecting tubing, drapes not soaked with bodily fluids) used within arthroscopy could be processed as clean waste and recycled without harm to those involved in the processing. This has both environmental and economic implications given the cost of waste management for clean and contaminated waste.

The waste produced over the four hospital sites varied enormously, with one key factor being whether the site used reusable or disposable drapes and gowns. There is work currently underway, via the Royal College of Surgeons of England Green Surgery Oversight Committee, to identify ways in which surgery can be made greener and more environmentally friendly. One of these aspects is assessing waste, including whether medical textile waste, such as gowns and drapes, can be minimised without incurring patient harm.

The costs of waste disposal varied widely depending on the contractor, contract negotiated and the methods of waste disposal used. Site A had on-site processing by incineration with harvesting of the energy generated. Because they used reusable drapes and gowns, the quantity of waste was less at this site. Site B utilised a combination of off-site processing by incineration with harvesting of the energy generated alongside landfill options. Sites C and D used landfill for waste processing. Standardisation of contracts may reduce variability between hospitals with regards to the cost of waste processing.

Procurement also has a role to play because they source the items that we, as clinicians, use. There has already been some work on the multipacks used in the operating theatre. One study in the United States concerning wide-awake hand surgery cases had clinicians involved in slimming down what was in the multipack. The study reported a 13% reduction in waste per case and a cost saving of $125.^19^ A further unit removed 15 items from their disposable plastic pack and 7 from the hand pack with an estimated annual saving of US$17,381.05 in the unit from these changes alone.^20^ Putting this into context, in 2016, the American Society for Surgery of the Hand estimated that there were approximately 2,000 active hand surgeons in the United States; if each were to do 100 ‘green’ cases a year, there would be a cost saving of $2.13 million and a decrease in 506 tons of waste.^21^

### Study limitations

This study did not assess waste created by anaesthetic procedures. In addition, only a small data set was examined with five patients for each of the two procedures at each site. Further research should be directed at assessing waste production from other orthopaedic procedures and consideration given to waste produced by anaesthetics. One study assessing waste from primary hip arthroplasty had similar findings: excess waste being produced and not being placed in the correct waste streams.^22^ Some types of plastic are more readily recycled than others, which cannot be recycled. Further investigation into the type of plastic disposed of during procedures would be valuable to assess whether we are using appropriate plastic types to create a greener operating environment. Finally, more work is required to assess the cost of laundering reusable gowns and the environmental impact this has compared with disposable gowns and drapes.

## Conclusions

To conclude, the data collected during this study demonstrated significant variability in waste production between hospital sites and a highly variable cost for waste disposal dependent upon the nature of the contract and method of disposal used by the waste management company. Clinicians should be working with hospital management and procurement to create an environmentally more sustainable operating theatre with consideration given to the methods in which waste is managed. At a national level, consideration should be given to both the guidance for and procurement of appropriate products such that waste can be efficiently recycled or disposed of with attention paid to environmentally sustainable methods.
